# Reply to Comment “Assessing Porous Media Permeability in Non-Darcy Flow: A Re-Evaluation Based on the Forchheimer Equation”

**DOI:** 10.3390/ma13112544

**Published:** 2020-06-03

**Authors:** A. P. G. Castro, T. Pires, J. E. Santos, B. P. Gouveia, P. R. Fernandes

**Affiliations:** IDMEC, Instituto Superior Técnico, Universidade de Lisboa, 1649-004 Lisbon, Portugal; tiago.a.h.v.pires@tecnico.ulisboa.pt (T.P.); jemidiocsantos@gmail.com (J.E.S.); bgouveia@tecnico.ulisboa.pt (B.P.G.); paulo.rui.fernandes@tecnico.ulisboa.pt (P.R.F.)

In the name of the authors of the paper “Permeability versus Design in TPMS Scaffolds”, published in *Materials* in 2019 and already counting six citations in Google Scholar to date, I would like to formally respond to the comment manuscript now entitled “Assessing Porous Media Permeability in Non-Darcy Flow: A Re-Evaluation Based on the Forchheimer Equation”.

We would like to thank the authors of the comment manuscript for contributing to the enhancement of the scientific discussion, and also to acknowledge the comments of the external reviewer.

Our paper is part of a much wider project on the design of biomedical scaffolds. The objective of the paper was to evaluate the biomechanical behavior of triply periodic minimal surfaces (TPMS) scaffolds for bone tissue engineering applications, namely their fluid permeation capabilities.

To this end, we combined experimental permeability tests and computational models (using the finite element method) to perform a qualitative behaviour comparison, the Gyroid (SG) scaffolds being the more permeable ones, with the Schwartz D (SD) being the less permeable. We agree that Forchheimer’s law is useful in this context, but the permeability values calculated by employing Forchheimer’s law are not significantly different from the ones previously calculated with Darcy’s law, which means that the conclusions of our work are not altered by using Darcy or Forchheimer permeabilities. This is still not acknowledged in the comment manuscript.

It is our final opinion that the manuscript should not be published as a comment to our paper. It should instead be published as a separate technical paper that uses our data to re-calculate permeability with a different approach (Forchheimer’s law). 

